# Discovery of a Highly Conserved Peptide in the Iron Transporter Melanotransferrin that Traverses an Intact Blood Brain Barrier and Localizes in Neural Cells

**DOI:** 10.3389/fnins.2021.596976

**Published:** 2021-06-02

**Authors:** Chaahat S. B. Singh, Brett A. Eyford, Thomas Abraham, Lonna Munro, Kyung Bok Choi, Mark Okon, Timothy Z. Vitalis, Reinhard Gabathuler, Chieh-Ju Lu, Cheryl G. Pfeifer, Mei Mei Tian, Wilfred A. Jefferies

**Affiliations:** ^1^Michael Smith Laboratories, University of British Columbia, Vancouver, BC, Canada; ^2^The Vancouver Prostate Centre, Vancouver General Hospital, Vancouver, BC, Canada; ^3^Centre for Blood Research, University of British Columbia, Vancouver, BC, Canada; ^4^The Djavad Mowafaghian Centre for Brain Health, University of British Columbia, Vancouver, BC, Canada; ^5^Department of Medical Genetics, University of British Columbia, Vancouver, BC, Canada; ^6^Department of Neural and Behavioral Sciences and Microscopy Imaging Core Lab, Pennsylvania State College of Medicine, Hershey, PA, United States; ^7^Department of Chemistry, University of British Columbia, Vancouver, BC, Canada; ^8^Bioasis Technologies Inc., Guilford, CT, United States; ^9^King’s College London, London, United Kingdom; ^10^Department of Urologic Sciences, University of British Columbia, Vancouver, BC, Canada; ^11^Department of Microbiology and Immunology, University of British Columbia, Vancouver, BC, Canada; ^12^Department of Zoology, University of British Columbia, Vancouver, BC, Canada

**Keywords:** blood-brain barrer, MTfp, drug delivery and targeting, peptide transport, neuronal targeting, microglial targeting, glial targeting, melanotransferrin

## Abstract

The blood-brain barrier (BBB) hinders the distribution of therapeutics intended for treatment of diseases of the brain. Our previous studies demonstrated that that a soluble form of melanotransferrin (MTf; Uniprot P08582; also known as p97, MFI2, and CD228), a mammalian iron-transport protein, is an effective carrier for delivery of drug conjugates across the BBB into the brain and was the first BBB targeting delivery system to demonstrate therapeutic efficacy within the brain. Here, we performed a screen to identify peptides from MTf capable of traversing the BBB. We identified a highly conserved 12-amino acid peptide, termed MTfp, that retains the ability to cross the intact BBB undigested, distribute throughout the parenchyma, and enter endosomes and lysosomes within neurons, astrocytes and microglia in the brain. This peptide may provide a platform for the transport of therapeutics to the CNS, and thereby offers new avenues for potential treatments of neuropathologies that are currently refractory to existing therapies.

## Introduction

Many neurological diseases of the central nervous system (CNS) remain untreatable because the blood-brain barrier (BBB) excludes efficacious drugs from entering the brain (Rapoport, 2000; Cressant et al., 2004; Pardridge, 2007a; Banks, 2016). Methods developed to enhance the delivery of drugs to treat diseases in the brain often fail to provide significant improvements to long-term survival (Braasch et al., 2004; Kleinschnitz et al., 2010; Radermacher et al., 2013).

Several studies describe examples of antibodies conjugated to drugs that can cross the BBB as a result of their interaction with specific receptors (Jefferies et al., 1984), that suggests that drugs conjugated to the natural, endogenous ligands for these specific receptors may be of value in the ferrying therapeutic cargo to the brain (Boado et al., 2007). In order to address this, we have investigated the expression and distribution of ligands and receptor molecules on brain capillary endothelium (Food et al., 1994; Rothenberger et al., 1996; Demeule et al., 2002; Moroo et al., 2003), with the goal that these may provide novel routes of entry into the brain.

Melanotransferrin (MTf, Uniprot P08582; also known as p97, MFI2 and CD228) is an iron-binding glycoprotein belonging to the transferrin (Tf) family of molecules. MTf shares a 40% protein sequence identity with human lactoferrin and Tf (Rose et al., 1986), and is highly conserved across various species (McNagny et al., 1996). MTf is one of the oldest members in this family of proteins, dating back to more than 670 million years, and may have split from serum Tf soon after the duplication of the N and C terminal lobes, suggesting an indispensable role for MTf in iron metabolism (Lambert et al., 2005). In many species, from insects to mammals, alternative RNA splicing yields both secreted, soluble form (sMTf) and glycosylphosphatidylinositol (GPI)-anchored forms of MTf (gpiMTf) (Kennard et al., 1995; Moroo et al., 2003; Yang et al., 2004). Using isothermal titration calorimetry and differential scanning calorimetry (Creagh et al., 2005), we determined that, unlike Tf, MTf binds only one molecule of iron with an apparent binding affinity constant of 4.4 × 10^17^ M^–1^, which is an affinity intermediate between the binding constants of iron to the N- and C-lobes of Tf measured under the same conditions, suggesting that MTf may successfully compete for iron *in vivo* (Rose et al., 1986; Lin et al., 1994; Kennard et al., 1995; Demeule et al., 2002; Moroo et al., 2003). The three dimensional structure of MTf has only recently been determined (Hayashi et al., 2021), and while it is useful to draw comparisons with available crystal structure data for Tf (in both its apo and holo states), it is clear that MTf and Tf have notable structural differences, most notably MTf exists as a soluble and membrane-bound form, while Tf is only found in a soluble form (Baker et al., 1992; Alemany et al., 1993; Food et al., 1994). The gpiMTf form can transport iron into mammalian cells (Kennard et al., 1995), while sMTf is able to efficiently cross the BBB and deliver iron to the CNS (Moroo et al., 2003). The latter result is particularly noteworthy as no other Tf family member has been reported to possess significant BBB transcytotic function (Kennard et al., 1995; Demeule et al., 2002; Moroo et al., 2003; Thom et al., 2018a). A broadly-specific receptor (low-density lipoprotein receptor-related protein) is capable of binding plasminogen-MTf complexes and appears to act as a receptor for MTf (Demeule et al., 2003).

We previously created a recombinant soluble form of MTf (Yang et al., 2004) analogous to sMTf that is an effective carrier for delivery of drug conjugates across the BBB into the brain (Demeule et al., 2002; Moroo et al., 2003; Karkan et al., 2008). This form of sMTf is actively transported across the BBB, by receptor mediated transcytosis at rates 10–15 times higher than those obtained with either serum transferrin (Tf) or lactoferrin (Lf) (Demeule et al., 2002). In addition, therapeutics conjugated to sMTf, can be shuttled across the BBB and were among the first BBB targeting delivery system to demonstrate therapeutic efficacy within the brain. For example, mice suffering from otherwise inaccessible brain tumors were treated with sMTf-drug or sMTf-antibody conjugates resulting in reduction of tumor growth (Karkan et al., 2008; Gabathuler et al., 2013; Nounou et al., 2016). Thus, sMTf is established as an endogenous protein with clear potential as a BBB drug delivery vehicle, however, MTf is relatively large (738 residue, ∼80 kDa) (Rose et al., 1986) and this may limit its versatility as a “trojan horse” in brain delivery.

The variability in conformation, glycosylation, anchoring and metal binding, presents potential complications to the use of MTf as a robust, reproducible, clinically useful drug delivery vehicle. Therefore, to improve upon the utility of sMTf as a drug-delivery vector, we fragmented the sMTf protein in order to identify peptides that retains the capability of carrying molecular cargo across the BBB. Here we report the identification of a fully functional, 12 amino acid derived from sMTf termed MTfp, that penetrates the brain after peripheral injection. We have previously reported that MTfp is capable of delivering a protein-based interleukin 1 receptor antagonist across the BBB and effectively ameliorates neuropathic pain in a preclinical model (Thom et al., 2018b). Furthermore we recently reported MTfp can act as a nanomule to deliver NOX4-specific siRNA to the brain that attenuate ischemic stroke (Eyford et al., 2021). Here we present comprehensive documentation on the discovery MTfp using a combination of bioinformatics and structural protein studies, its efficacy of transport into the brain of live animals, and its localization within intracellular compartments in microglia, neurons, and astrocytes within the brain parenchymal cells using microscopy.

## Materials and Methods

### Animal Experiments

All protocols and procedures involving the care and use of animals in these studies reviewed and approved by the University of British Columbia Animal Care Committee, which operates under the Canadian Council for Animal Care. All studies involving live animals complied with ARRIVE guidelines (Percie du Sert et al., 2020).

### Purification and Fragmentation of MTf

Recombinant human sMTf was expressed and purified at the University of British Columbia, as described previously (Hegedus et al., 1999; Yang et al., 2004). To perform trypsin digestion, 200 μL of MTf (10 mg/mL) were diluted in 1 mL of 0.15 M Tris, 6 M guanidine, 10 mM dithiothreitol, pH 9. The sample was heated at 60°C for 15 min and cooled to room temperature. The solution was further diluted with 1.8 mL of 0.15 M Tris, pH 9. Forty μg of sequencing grade modified trypsin (Cat. #V5111, Promega) were dissolved in 400 μL of 0.15 M Tris and added to the reaction mixture (1:50 trypsin to MTf ratio). The sample was incubated at 37°C for 20 h, lyophilized and stored at −20°C. The digest was dissolved in 5 mL of 0.1% formic acid and the tryptic peptide mixture was cleaned using Sep-Pack C8 cartridges (Cat. #WAT036775, Waters). The tryptic peptides were lyophilized and stored at −20°C until transcytosis assays were performed.

### Mass Spectrometry for Peptide Identification

Peptide identification was performed by MRM Proteomics (Victoria, BC). Lyophilized supernatants from the *in vitro* BBB experiments were rehydrated with 2% acetonitrile, 0.1% formic acid. The samples were resolved by liquid chromatography at 0.4 mL/min, over 43 min, using an Agilent Eclipse Plus C18 (150 × 2.1 mm, 1.8 μm) column. The eluates were analyzed on an Agilent 6490 QQQ mass spectrometer in positive mode, controlled by Agilent’s MassHunter Workstation software (version B.04.01). All acquisition methods used the following parameters: 3,500 V capillary voltage, 250°C sheath gas temperature at 11 L/min sheath gas flow. The electron multiplier had a 400 V offset, the nebulizer was set to 30 p.s.i., and the first and third quadrupoles were set to unit resolution. A default fragmentor voltage and cell accelerator voltage of 380 and 5 V, respectively. The maximum cycle time was 352.5 ms. All data was processed using Agilent Quantitation software (v.B.04.01) with automatic peak detection and smoothing Gaussian width of 5. All integrated peaks were manually inspected to ensure correct peak detection and integration. For accuracy, peaks with intensity less than 50 counts and/or intra-ion pair retention time difference greater than 0.02 min were not quantitated. Quantitation was achieved by spiking a predetermined, optimized mixture of stable isotope standard peptides into the relevant experiment: YYDYSGAFR = 500 fmol/μL; DSSHAFTLDELR = 2 pmol/μL; ADVTEWR = 100 fmol/μL; VPAHAVVVR = 200 fmol/μL; ADTDGGLIFR = 200 fmol/μL. The Uniprot file human MTF used for identification was P08582^1^ and the peptide peak lists were submitted to a Mascot 2.3 server against the Uniprot-Swissprot database. The peak areas were calculated for the top three peptides for each protein detected with high confidence.

### *In vitro* BBB Model and Transcytosis Assays

Work involving peptide transcytosis through the *in vitro* BBB model was performed by Cellial Technologies (Lens France). The *in vitro* model of the BBB consists of primary bovine brain capillary endothelial cells grown to confluence on collagen coated; polycarbonate transwell inserts (0.4 μm pore size, 24 mm diameter, Cat. #3412, Corning) and supported by primary rat glial cells. Under these conditions, endothelial cells retain endothelial markers (factor VIII –related antigen, non-thrombogenic surface, production of prostacyclin, angiotensin converting enzyme activity) and characteristics of the BBB (presence of tight junctions and P-glycoprotein, paucity of pinocytotic vesicles, monoamine oxidase activity, γ-glutamyl transpeptidase activity and 500–800 Ω/cm^2^ electrical resistance) (Meresse et al., 1989; Dehouck et al., 1990; Fenart et al., 1998).

Primary cultures of glial cells were isolated from newborn rat cerebral cortex (Booher and Sensenbrenner, 1972). After the meninges had been cleaned off, the brain tissue was forced gently through a nylon sieve. DMEM supplemented with 10% (v/v) fetal bovine serum, 2 mM glutamine and 50 μg/mL of gentamicin was used for the dissociation of cerebral tissue and development of glial cells. The glial cells were plated at a concentration of 1.25 × 10^5^ cells/mL in six-well plates and incubated at 37°C in 5% CO_2_. The medium was changed twice a week. Three weeks after seeding, cultures of glial cells become stabilized.

Primary bovine brain capillary endothelial cells were cultured on gelatin-coated Petri dishes in DMEM supplemented with 10% (v/v) calf serum, 10% (v/v) horse serum, 2 mM glutamine and 50 μg/mL gentamicin. One ng/mL of basic fibroblast growth factor was then added every other day. Under these conditions, endothelial cells form a confluent monolayer in 12 days. Transwell inserts were coated, on the upper side, with rat-tail collagen prepared according to the method of Bornstein (Bornstein, 1958). Confluent endothelial cells were trypsinized and plated on the upper side of the filters at a density of 4 × 10^5^ cells per insert.

Lucifer Yellow (LY), at 20 μM, was used as a paracellular marker allowing the evaluation of the integrity of the BBB. Ringer-HEPES buffer (150 mM NaCl; 5.2 mM KCl; 2.2 mM CaCl2; 0.2 mM MgCl2•6H_2_0; 6 mM NaHCO_3_; 5 mM HEPES; 2.8 mM glucose) was added to the lower compartment (abluminal side) of a six-well plate (3 mL per well). One mL Ringer-HEPES buffer containing the trypsin-digested MTf or synthetic peptide (0.25 mg/mL), in co-incubation with LY, was placed in the upper compartment (luminal side). Incubations were performed on a rocking platform at 37°C for 2 h. Experiments were performed in triplicate with filters containing a confluent monolayer of endothelial cells or in triplicate with empty filters coated only with collagen. At the end of the incubation period, aliquots of abluminal and luminal liquid were collected. Detection of LY fluorescence was performed using a fluorescence counter (Fluoroskan Ascent, Thermolab Systems) to ensure BBB integrity. Supernatants from the luminal and abluminal compartments were lyophilized and stored at −80°C until peptide analysis by mass spectrometry.

### Synthesis of MTfp Conjugates

MTfp (DSSHAFTLDELRYC), reversed MTfp (revMTfp; RLEDLTFAHSSDYC), and the positive control for BBB transport, RVGp (YTIWMPENPRPGTPCDIFTNSRGKRASNGYC) were synthesized by Anaspec (Fremont, United States) and conjugated to Cy5. Each peptide was synthesized with addition “YC” residues on the C terminus to facilitate fluorescent labeling. After the reaction, the crude reaction mixture was purified using semi-preparative reverse phase C18 chromatography. The fractions containing the blue-colored product were collected, pooled, lyophilized and stored at −80°C.

### 3D Fluorescence Microscopy Imaging

Three sets of 3D fluorescence microscopy experiments were performed. The first set measured the BBB penetration of Cy5 tagged MTfp using DeltaVision Elite integrated with Huygens Deconvolution Software (GE, United States). As an extension of this, a set of sections were prepared to assess the cellular localization of the Cy-5 tagged MTfp in the CNS. The second set of experiments addressed the localization of Cy-5 tagged MTfp in various cell types and subcellular objects using the Leica SP8 X system (Leica, Germany).

To show that MTfp can cross the BBB, PBS, RVGp-Cy5, revMTfp-Cy5 and MTfp-Cy5 were injected IV into 6–8 week-old, female CD-1 mice (0.5 mM in PBS, 0.1 mL/mouse). Briefly, CD-1 female mice (19.4–25.6 g body weight on day of dosing, 3 per group) were injected once with PBS or one of the peptides. Two hours post injection, mice received a second IV injection of tomato lectin conjugated to FITC (100 μg/mouse, 0.1 mL). Ten minutes later, the animals were terminally anesthetized with an IP injection of 0.2 mL ketamine (200 mg/kg) and Rhompun (20 mg/kg). The bloodstream was flushed by intracardiac perfusion for 10 min at a flow rate of 1 mL/min with heparinized saline (0.9% NaCl, 100 U/mL heparin).

The brains were removed and fixed with 4% paraformaldehyde overnight and then transferred into PBS+0.01% sodium azide and stored at 4°C. The brains were embedded in 4% agarose, fixed onto the microtome stage and sectioned (20 μm) at 4°C. The sections were stained with DAPI and then mounted on microscopic slides. Glass coverslips were mounted on the sections using Prolong Gold antifade reagent. In another experiment, to assess the localization of the peptide in the CNS, brains from mice injected with either PBS or Cy5-MTfp followed by FITC- tomato lectin. The brains were collected and processed as stated above. These were then incubated with antibodies targeted against neurons (NeuN; ab177487), microglia (TMEM; ab209064), astrocytes (GFAP; ab7260), lysosomes (LAMP-1; sc-19992-AF546) and endosomes (EEA-1; sc-137130), secondary antibody staining was done for unconjugated primary antibodies with Alexa fluor 680 goat anti-mouse IgG (cat# A21058), Alexa fluor 680 goat anti-rabbit IgG (Cat #A21076) and then counter-stained with DAPI, cover slipped, imaged and quantitatively analyzed to determine the extent of localization of various cell types and subcellular objects with MTfp.

The microscopy experiments, which quantitatively assessed the cellular location of MTfp within the brain, were performed using Leica SP8 X system (Leica, Germany). 3D confocal images were acquired with a Leica AOBS SP8 laser scanning confocal microscope (Leica, Heidelberg, Germany) using a high-resolution Leica 63X/1.4 or 40X/1.3 Plan-Apochromat oil immersion objective lens. Excitations were performed using either diode or tunable white light laser sources. All images and spectral data (except DAPI) were generated using the highly sensitive HyD detectors (with gated option) in de-scanned mode. The backscattered emission signals from the sample were delivered through the tunable filter (AOBS), the detection pinhole, spectral dispersion prism, and finally to the PMT/HyD detectors. For 3D image data set acquisition, the excitation beam was first focused at the maximum signal intensity focal position within the brain tissue sample and the appropriate HyD gain levels were then selected to obtain the pixel intensities within range of 0–255 (8-bit images) using a color gradient function. The beginning and end of the 3D stack were set based on the signal level degradation. Series of 2D images for a selected 3D stack volume were then acquired with 1,024 × 1,024 pixels. The 3D stack images with optical section thickness (z-axis) of approximately 0.3 μm were captured from tissue volumes.

Spectral measurements to confirm the presence of Cy5 signal in the brain parenchyma were also performed using 32-channel Nikon Spectral Detector integrated with Nikon A1 MP+ Multi- Photon Microscope system (Nikon Instruments, New York). The laser used to produce the fluorescence emission from Cy5 was a mode-locked femto-second Spectra-Physics InSight DS femtosecond single-box laser system with automated dispersion compensation tunable between 680 and 1,300 nm (Spectra-Physics, Mountain View, CA). The laser output was attenuated using AOTF and the average power was consistently maintained below the damage threshold of the samples. The power attenuated laser was directed to a Nikon scan head coupled with Nikon upright microscope system (Nikon Instruments, New York). The laser beam was then focused on the specimen through a high numerical aperture, low magnification, long working distance, water immersion objective, CFI75 Apo Water 25X/1.1 LWD 2.0 mm WD. The backscattered emission from the sample was collected through the same objective lens. Nikon NIS Element Software was used for the image acquisition.

For each tissue volume reported here, z-section images were compiled and finally the 3D image restoration was performed using VOLOCITY software (Perkin Elmar, United Kingdom). The volume estimation was performed on the 3D image data sets recorded from four or more cortical area of brain tissue samples. Algorithms were developed to automate the quantification of the test materials and blood capillaries, and to accurately quantify test materials localized in blood capillaries vs. brain parenchyma. In these procedures, a noise removal filter (kernel size of 3 × 3) was used to remove the noise associated with the images. To define the boundary between the objects (for instance, blood capillaries) and the background, the lower threshold level in the histogram was set to exclude all possible background voxel values. The sum of all the voxels above this threshold level is determined to be the volume. Fields from each experimental group were pooled.

### Statistics

#### For Microscopy Images

ANOVA was used to assess the volume fractions (for instance, MTfp-Cy5) in the brain parenchyma (Prism 6, Graphpad, La Jolla, United States). The level of significance was *p* < 0.05 for the ANOVA and the *p*-values were corrected for multiple comparisons in the *post-hoc* analysis.

### Nuclear Magnetic Resonance

All experiments were performed in a Bruker Ascend 850 MHz magnet at the University of British Columbia. Data were processed and analyzed using the programs TopSpin (BRUKER Ltd.) and Sparky (Goddard and Kneller) respectively. Proton correlation (COSY), total correlation (TOCSY), nuclear Overhauser effect (NOESY) spectroscopy as well as proton-carbon heteronuclear single quantum correlation (HSQC) and heteronuclear multiple bond correlation (HMBC) spectroscopy were performed on synthetic, 5 mM DSSHAFTLDELR peptide in 25 mM sodium phosphate buffer (pH 6.5) with 5% D_2_O at 10, 20, 30, and 37°C. Spectral assignment was performed manually and chemical shift data were assessed by MICS (Motif Identification from Chemical Shifts) software (Shen and Bax, 2012).

### Virtual Crystal

The crystal structure of MTf was only determined recently (Hayashi et al., 2021), after the present study was completed. *In silico* homology models were generated using the Robetta structure server (Baker_Lab) (data not shown) and compared to published crystals structures of human Tf (Wally et al., 2006; Wally and Buchanan, 2007).

## Results

### Identification of MTfp

**FIGURE 1:**
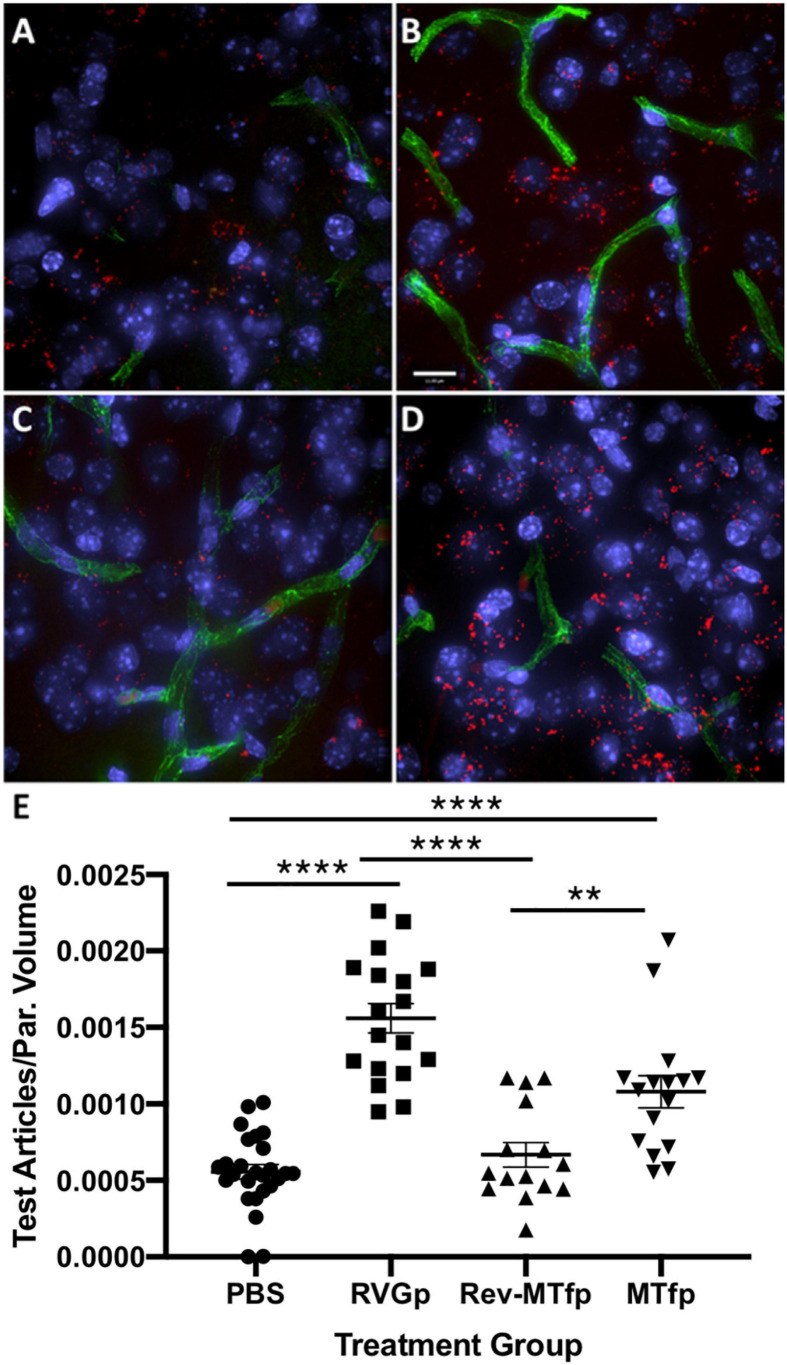
Representative deconvolved images showing localization of MTfp in the brains of mice. Cell nuclei are blue (DAPI) and capillaries are green (FITC). **(A)** Fluorescence (red) in the brain for a mouse treated with PBS (i.e., background fluorescence), **(B)** Cy5 fluorescence in the brain after IV injection with RVGp-Cy5, **(C)** Cy5 fluorescence in the brain after IV injection of revMTfp-Cy5, **(D)** Cy5 fluorescence in the brain after IV injection of MTfp-Cy5, **(E)** brain distribution of Cy5 in wild type mice, where values indicate total volume of Cy5 fluorescence in each tissue normalized to tissue volume (VTA_*B*__PV_ in Supplementary Table 1). Data are shown as individual points with the error bars indicating means ± SEM (*n* = 3, 3 sections/animal and 4–11 fields of view per section). **p < 0.01, ****p < 0.0001.

**FIGURE 2:**
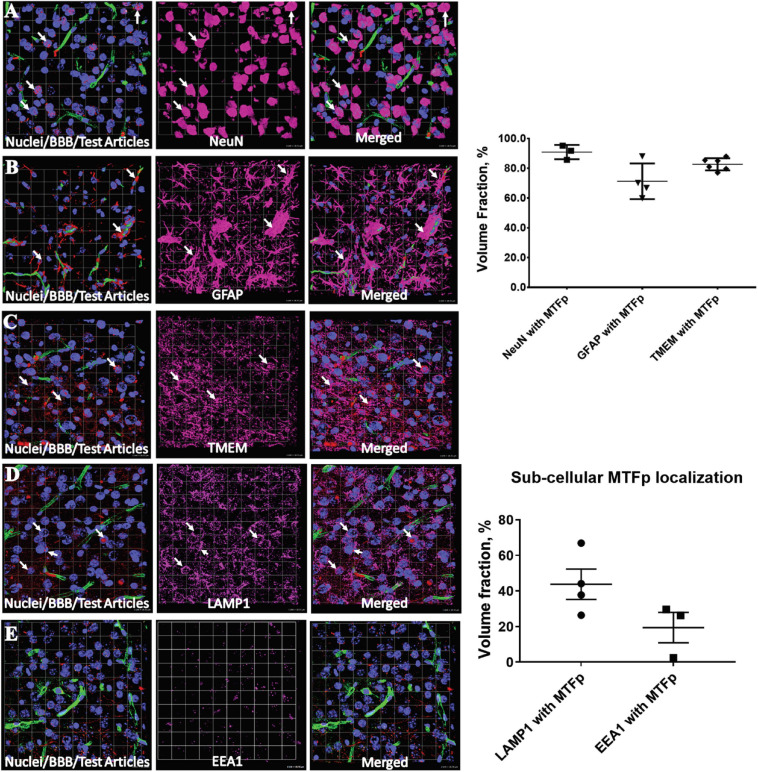
Representative 3D confocal images showing localization of various cell types and subcellular objects with MTfp in the mouse brain sections. Cell nuclei are in blue color (DAPI) and BBBs are in green color (FITC). (**A–C)** Cellular localization of MTFp and **(A–E)** subcellular localization of MTFp. **(A)** Localization of neuronal marker NeuN (Pink) with MTfp (Red), **(B)** localization of Astrocytes marker GFAP (Pink) with MTfp (Red), **(C)** localization of Microglia marker TMEM (Pink) with MTfp (Red), **(D)** localization of Lysosomal marker LAMP1 (Pink) with MTfp (Red), **(E)** localization of Endosomal marker EEA1 (Pink) with MTfp (Red). White arrows indicate co-localization of MTfp (red) and cells (pink). The graphs represent the extent of localization of various cell types and subcellular objects with MTfp in the CNS as a percentage of fluorescence volume fraction. Each animal is presented as an individual data point in the graph with the error bars indicating mean ± SEM.

In order to identify a fragment of sMTf, that retains the ability to cross the BBB, purified, recombinant human sMTf was digested with either hydroxylamine (NH_2_OH), cyanogen bromide (CNBr) or trypsin. The resulting fragments were placed on the luminal side of an *in vitro* bovine BBB model and, after 2 h, the presence of MTf fragments on the abluminal side was assessed by SDS-PAGE (NH_2_OH and CNBr) and mass spectrometry (all digestions). No fragments from the CNBr or NH_2_OH digests were found on the abluminal side of the BBB model (data not shown). However, 50 potential tryptic peptides were tested (Table 1), and five were identified in the abluminal medium of the *in vitro* BBB model: DSSHAFTLDELR; ADTDGGLIFR; VPAHAVVVR; ADVTEWR; and YYDYSGAFR. To determine relative transport efficiencies, these five peptides were synthesized and retested individually in the same BBB model system. Quantitation of transcytosis was achieved by spiking known concentrations of stable isotope labeled peptides prior to mass spectrometry. The most efficiently transcytosed peptide (DSSHAFTLDELR, MTf460-471, now known as MTfp, or commercially as xB^3^ ^TM^ peptide) was selected as the most promising candidate for use as a BBB transport vector (Table 2). MTfp transit across the BBB *in vivo* was tested in mice by injecting a MTfp-Cy5 conjugate intravenously (IV), followed by visualization and quantification by 3D deconvolution fluorescence microscopy (Figure 1 and Supplementary Table 1). Approximately two times greater fractional fluorescence was measured in the brain parenchyma of MTfp-Cy5 injected wild type (WT) mice when compared to control mice injected with PBS or reversed MTfp-Cy5 (revMTfp-Cy5). Interestingly, MTfp penetrated the BBB with efficiency statistically similar to that of a larger, known brain-targeting peptide derived from a rabies virus glycoprotein (RVGp). Next, the MTfp was shown to colocalize in neurons, microglia and astrocytes in the CNS. As shown in Figure 2, we demonstrated that 90% of the neurons expressing NeuN co-labeled with MTfp; 70% of astrocytes expressing GFAP co-labeled with MTfp; and 80% of microglia expressing TMEM co-labeled with MTfp. The subcellular markers investigated show that 40% of the MTfp is found in the lysosomes and approximately 20% is found in the early endosomes. This data demonstrates for the first time that MTfp injected IV, is widely dispersed in the brain and it is taken up into intracellular organelles.

**TABLE 1:** Soluble melanotransferrin tryptic peptides at 3 micron pore size.

Tryptic Peptide Sequence (SEQ ID NO:)	Ablum 120:Area	Lum 120:Area	Ablum 120 conf	lonScore Ablum 120	Exp Value Ablum 120	Lum 120conf	lonScore Lum 120	Exp Value Lum 120
LFSHEGSSFQMFSSEAYGQK (SEQ ID NO:55)	1.28E+09	4.55E+09	High	115	2.50E-11	High	130	8.40E-13
HTTVFDNTNGHNSEPWAAELR (SEQ ID NO:56)	1.28E+09	1.05E+10	High	106	2.80E-10	High	103	5.80E-10
HTTVFDNTNGHNSEPWAAELR (SEQ ID NO:56)	7.04E+09	1.92E+10	High	101	9.50E-10	High	109	1.40E-10
AVSDYFGGSCVPGAGETSYSESLCR (SEQ ID NO:57)	5.49E+08	5.51E+09	High	101	2.80E-10	High	125	1.20E-12
NYPSSLCALCVGDEQGR (SEQ ID NO:58)	7.34E+07	6.15E+08	High	100	6.60E-10	High	111	6.40E-11
TLPSWGQALLSQDFELLCR (SEQ ID NO:59)	2.25E+06	1.94E+09	High	94	5.10E-09	High	133	6.80E-13
AQDLFGDDHNKNGFK (SEQ ID NO:15)	9.09E+08	5.40E+08	High	87	2.40E-08	High	72	7.10E-07
CLAEGAGDVAFVK (SEQ ID NO:60)	2.20E+09	4.38E+09	High	87	3.10E-08	High	92	7.90E-09
MFDSSNYHGQDLLFK (SEQ ID NO:61)	9.62E+08	2.06E+09	High	86	2.50E-08	High	81	7.20E-08
ADTDGGLIFR (SEQ ID NO:10)	1.59E+10	1.11E+10	High	82	8.50E-08	High	82	9.10E-08
LFSHEGSSFQMFSSEAYGQK (SEQ ID NO:55)	1.94E+08	1.06E+09	High	81	5.50E-08	High	104	3.20E-10
MFDSSNYHGQDLLFK (SEQ ID NO:61)	5.67E+09	1.73E+10	High	79	1.40E-07	High	79	1.50E-07
MFDSSNYHGQDLLFK (SEQ ID NO:61)	3.22E+07	1.01E+08	High	79	1.10E-07	High	77	1.60E-07
CGDMAVAFR (SEQ ID NO:ll)	3.58E+09	7.79E+09	High	76	1.50E-07	High	79	7.30E-08
GDSSGEGVCDKSPLER (SEQ ID NO:6)	1.93E+09	5.08E+08	High	74	3.10E-07	High	82	4.20E-08
AQDLFGDDHNKNGFK (SEQ ID NO:15)	4.27E+08	7.66E+07	High	74	3.80E-07	Medium	28	1.50E-02
CGDMAVAFR (SEQ ID NO:ll)	4.54E+08	2.20E+08	High	71	3.40E-07	High	79	5.50E-08
LFSHEGSSFQMFSSEAYGQKDLLFK (SEQ ID NO:62)	1.30E+07	8.27E+07	High	70	1.40E-06	High	33	6.00E-03
RDSSHAFTLDELR (SEQ ID NO:63)	1.66E+09	5.02E+09	High	68	2.70E-06	High	80	1.60E-07
AQDLFGDDHNK (SEQ ID NO:64)	3.69E+09	1.08E+09	High	63	4.50E-06	High	55	2.60E-05
LSVMGCDVLK (SEQ ID NO:65)	2.43E+09	1.07E+10	High	62	9.70E-06	High	53	8.40E-05
SEDYELLCPNGAR (SEQ ID NO:14)	2.52E+08	1.25E+08	High	62	3.90E-06	High	49	8.00E-05
EAGIQPSLLCVR (SEQ ID NO:66)	8.66E+08	1.99E+09	High	60	1.20E-05	High	59	1.40E-05
SSHVTIDTLKGVK (SEQ ID NO:4)	1.12E+08	4.73E+07	High	60	8.50E-06	High	57	1.30E-05
WCATSDPEQHK (SEQ ID NO:2)	1.01E+09	1.37E+08	High	59	5.00E-06	High	57	7.90E-06
HTTVFDNTNGHNSEPWAAELR (SEQ ID NO:56)	0.00E+00	2.95E+07	High	55	2.90E-05	High	51	6.20E-05
LSVMGCDVLK (SEQ ID NO:65)	4.85E+08	3.08E+09	High	55	4.70E-05	High	53	8.10E-05
DSSHAFTLDELR (SEQ ID NO:13)	5.87E+09	1.03E+10	High	54	5.20E-05	High	59	1.70E-05
LCRGDSSGEGVCDK (SEQ ID NO:5)	2.64E+05	2.17E+05	High	52	2.90E-05	High	47	9.70E-05
SSHVTIDTLK (SEQ ID NO:67)	3.37E+09	1.74E+09	High	48	1.90E-04	High	43	6.80E-04
LKPEIQCVSAK (SEQ ID NO:12)	4.39E+09	1.00E+09	High	46	3.00E-04	High	45	4.20E-04
VPAHAVVVR (SEQ ID NO:9)	1.24E+08	3.48E+07	High	45	6.S0E-05	High	40	2.00E-04
ADVTEWR (SEQ ID NO:8)	1.05E+10	9.31E+08	High	44	4.80E-04	High	48	2.30E-04
RSSHVTIDTLK (SEQ ID NO:3)	1.64E+08	9.53E+07	High	43	6.90E-04	High	45	4.lOE-04
SEDYELLCPNGAR (SEQ ID NO:14)	2.10E+09	2.51E+08	High	42	4.80E-04	High	60	7.40E-06
WCVLSTPEIQK (SEQ ID NO:68)	1.09E+07	0.00E+00	Medium	37	4.20E-03			
YYDYSGAFR (SEQ ID NO:7)	6.41E+09	1.00E+09	Medium	31	3.80E-03	High	51	3.70E-05
GLLCDPNR (SEQ ID NO:69)	1.65E+09	1.97E+08	Medium	30	8.60E-03	Medium	29	1.20E-02
DSSHAFTLDELRGK (SEQ ID NO:70)	1.99E+07	0.00E+00	Low	26	5.20E-02			
GLLCDPNRLPPYLR (SEQ ID NO:71)	6.75E+09	2.32E+10	Low	25	3.20E-02	Low	19	1.40E-01
EHGLKPVVGEVYDQEVGTSYYAVAVVRR (SEQ ID NO:72)	1.70E+07	0.00E+00	Low	22	5.30E-02			
GLLCDPNRLPPYLR (SEQ ID NO:71)	1.09E+07	2.00E+08	Low	18	2.00E-01	Medium	26	3.lOE-02
CVGNSQERYYGYR (SEQ ID NO:73)	4.94E+06	0.00E+00	Low	18	1.30E-01			
CLVENAGDVAFVR (SEQ ID NO:74)	1.30E+08	5.49E+08	Low	16	4.lOE-01	High	72	1.20E-06
DSTS ELVPIATQTYEA WLGHEYLHAM K (SEQ ID NO:75)	1.55E+07	3.56E+08	Low	15	4.lOE-01	Low	11	1.00E+00
DSTS ELVPIATQTYEA WLGHEYLHAM K (SEQ ID NO:75)	0.00E+00	0.00E+00	Low	12	7.80E-01			
TLPSWGQALLSQDFELLCR (SEQ ID NO:59)	0.00E+00	0.00E+00				High	111	1.00E-10
LFSHEGSSFQMFSSEAYGQK (SEQ ID NO:55)	0.00E+00	0.00E+00				High	79	1.20E-07
IQAEQVDAVTLSGEDIYTAGK (SEQ ID NO:76)	0.00E+00	3.48E+06				High	75	4.30E-07
HSTVLENTDGK (SEQ ID NO:77)	0.00E+00	2.10E+07				High	66	2.70E-06
TVGWNVPVGYLVESGR (SEQ ID NO:78)	0.00E+00	9.38E+07				High	62	8.40E-06
LLNEGQR (SEQ ID NO:79)	0.00E+00	1.71E+07				High	43	4.30E-04
LFSHEGSSFQMFSSEAYGQKDLLFK (SEQ ID NO:80)	0.00E+00	0.00E+00				High	40	1.20E-03
ADTDGGLIFRLLNEGQR (SEQ ID NO:81)	0.00E+00	3.69E+07				High	40	1.50E-03
HTTVFDNTNGHNSEPWAAELR (SEQ ID NO:56)	0.00E+00	0.00E+00				High	38	1.20E-03

**TABLE 2:** Tryptic peptides of sMTf cross an *in vitro* BBB.

Peptide	Abundance in trypsin digest (fmol)	Luminal abundance (*T* = 120, fmol)	Abluminal abundance (*T* = 120, fmol)	Abluminal vs. luminal (%)	Abluminal vs. total (%)
DSSHAFTLDELRI	2.05E+04	1.62E+04	3.90E+02	2.41	1.90
ADTDGGLIFR	9.69E+04	7.19E+04	1.55E+03	2.16	1.60
VPAHAVWR	5.25E+03	3.20E+03	7.96E+01	2.49	1.52
ADVTEWR	6.09E+04	3.79E+04	5.02E+02	1.32	0.82
YYDYSGAFR	2.26E+04	8.43E+03	1.13E+01	0.13	0.05

*Molar abundances are normalized against compartment volumes. Fraction of peptide found in the abluminal compartment compared to the initial concentration is used as the measure of transcytosis efficiency. The discrepancy between Initial concentration and abluminal concentration plus luminal concentration is likely explained by a combination of peptide degradation and adhesion to the collagen matrix or cellular monolayer.*

### Structural Studies of MTfp

Structural studies were carried out to identify unique features of MTfp that enable it to retain the ability of the parent molecule to cross the BBB and also differentiate it from its phylogenetic cousins such as Tf and Lf, which do not efficiently cross the BBB. MTfp (DSSHAFTLDELR) is well-conserved through evolution (Table 3). There is perfect sequence identity among most primates and strong conservation among many other mammals. Divergence is typically restricted to the first five residues (DSSHA). Although the primary amino acid sequence of MTfp and the analogous Tf peptide (Tfp) are different, their location on their respective proteins and their secondary structures are predicted to be nearly identical. Both are displayed as surface-exposed amphipathic turns/loops between beta-strands. The hydrophobic residues of MTfp (Phe_465_, Leu_467_, and Leu_470_) are oriented toward the core of the protein, analogous to hydrophobic residues in Tfp, while the remaining residues appear to be solvent-exposed. In its protein context, both MTfp and Tfp are generally unstructured but anchoring by the hydrophobic residues seems to impart a slight helical character in the conserved C-terminal region (FTLDELR). Based on secondary structure comparison, it appears that the key differences between MTfp and Tfp are MTf His_463,_ which is absent in the shorter, 11 amino acid peptide of Tfp and the substitution of an asparagine in Tfp for glutamate in MTfp at position 469 in the putative helical region. It is likely that the polar orientation and slight helical conformation of MTfp are important for MTfp’s receptor binding capability. In the MTf protein context, this polarity would be maintained by repulsion between adjacent acidic residues and hydrophobic interactions with the core of the protein. However, these stabilizing hydrophobic interactions are absent when MTfp exists as a peptide in solution. It is possible that an entropic cost, needed to orient the free peptide into an appropriate receptor- binding conformation, may reduce receptor affinity and thus rates of transcytosis. However, we have shown that MTfp is capable of transcytosis, comparable to intact sMTf.

**TABLE 3:** Phylogenetic comparisons of MTfp homologues.

Common name	Species	Protein name	Accession #	Identity (%)	Sequence
Human	*Homo Sapien*	Melanotransferrin	NP_005920.2	100	DSSHAFTLDELR
Squirrel monkey	*Saimiri boliviensis boliviensis*	Melanotransferrin	XP_003926465.1	100	DSSHAFTLDELR
Bonobo	*Pan paniscus*	Melanotransferrin	XP_003806495.1	100	DSSHAFTLDELR
Chimpanzee	*Pan troglodytes*	Melanotransferrin	XP_003310242.2	100	DSSHAFTLDELR
Crab-eating macaque	*Macaca fascicularis*	hypothetical protein	EHH61865.1	100	DSSHAFTLDELR
Gibbon	*Nomascus leucogenys*	Melanotransferrin	XP_004089765.1	100	DSSHAFTLDELR
Baboon	*Papio anubis*	Melanotransferrin	XP_003895366.1	100	DSSHAFTLDELR
Rhesus macaque	*Macaca mulatta*	hypothetical protein	XP_001096034.2	100	DSSHAFTLDELR
Western lowland gorilla	*Gorilla gorilla gorilla*	Melanotransferrin	XP_004038326.1	100	DSSHAFTLDELR
Marmoset	*Callithrix jacchus*	Melanotransferrin	XP_005545367.1	100	DSSHAFTLDELR
Jerboa	*Jaculus jaculus*	Melanotransferrin	XP_004654417.1	92	DSSDAFTLDELR
Galago	*Otolemur garnettii*	Melanotransferrin	XP_003792792.1	92	DSSHSFTLDELR
Orangutan	*Pongo abelii*	Melanotransferrin	XP_002814515.1	92	DSSDAFTLDELR
Ground squirrel	*Ictidomys tridecemlineatus*	Melanotransferrin	XP_005341032.1	92	DSSYAFTLDELR
Rhinoceros	*Ceratotherium simum simum*	Melanotransferrin	XP_004424596.1	92	NSSHAFTLDELR
Alpaca	*Vicugna pacos*	Melanotransferrin	XP_006200994.1	83	NSSYAFTLDELR
Pika	*Ochotona princeps*	Melanotransferrin	XP_004578302.1	83	DSSYAFPLDELR
Flying fox	*Pteropus alecto*	Melanotransferrin	ELK17609.1	83	NSSYAFTLDELR
Bottlenose dolphin	*Tursiops truncatus*	Melanotransferrin	XP_004327245.1	83	NSSYAFTLDELR
Treeshrew	*Tupaia chinensis*	Melanotransferrin	XP_006156877.1	83	DSTHAFTVDELR
Chirrup	*Pantholops hodgsonii*	Melanotransferrin	XP_005966619.1	83	NSSYAFTLDELR
Domestic cat	*Felis catus*	Melanotransferrin	XP_003991825.1	83	NSSYAFTLDELR
Domestic cattle	*Bos taurus*	Melanotransferrin	NP_001179241.1	83	NSSYAFTLDELR
Domestic ferret	*Mustela putorius furo*	Melanotransferrin	XP_004806840.1	83	NSSYAFTLDELR
Giant panda	*Ailuropoda Melanoleuca*	Melanotransferrin	XP_002916802.1	83	NSSYAFTLDELR
Goat	*Capra hircus*	Melanotransferrin	XP_005675137.1	83	NSSYAFTLDELR
Mouse	*Mus musculus*	Melanotransferrin	NP_038928.1	83	DSSYSFTLDELR
Orca	*Orcinus orca*	Melanotransferrin	XP_004278827.1	83	NSSNAFTLDELR
Chinchilla	*Chinchilla lanigera*	Melanotransferrin	XP_005383209.1	83	DSSSAFTLNELR
Armadillo	*Dasypus novemcinctus*	Melanotransferrin	XP_004465950.1	83	DSSYAFTLDELW
Brown rat	*Rattus norvegicus*	Melanotransferrin	NP_001099342.1	83	DSSYSFTLDELR
Walrus	*Odobenus rosmarus divergens*	Melanotransferrin	XP_004391934.1	83	NSSSAFTLDELR
Prairie vole	*Microtus ochrogaster*	Melanotransferrin	XP_005344882.1	83	DSSYSFTLDELR
Domestic sheep	*Ovis aries*	Melanotransferrin	XP_004003067.1	83	NSSYAFTLDELR
Weddell seal	*Leptonychotes weddellii*	Melanotransferrin	XP_006742939.1	83	NSSYAFTLDELR
Bactrian camel	*Camelus ferus*	Melanotransferrin	XP_006179034.1	83	NSSYAFTLDELR
Wild boar	*Sus scrofa*	Melanotransferrin	XP_001926353.5	83	NSSYAFTLDELR
Yak	*Bos mutus*	Melanotransferrin	XP_005897887.1	83	NSSYAFTLDELR
African savanna elephant	*Loxodonta africana*	Melanotransferrin	XP_003412850.1	75	NSSYAFTMDELR
Chinese hamster	*Cricetulus griseus*	Melanotransferrin	ERE76419.1	75	DRSYSFTLDELR
Rabbit	*Oryctolagus cuniculus*	Melanotransferrin	NP_001075461.1	75	DSAYAFTVDELR
Degu	*Octodon degus*	Melanotransferrin	XP_004644626.1	75	DSSSAFNLNELR
Domestic dog	*Canis lupus familiaris*	Melanotransferrin	XP_005639711.1	75	NSSDAFSLDELR
Domestic guinea pig	*Cavia porcellus*	Melanotransferrin	XP_003477010.2	75	DSSSAFSLNELR
Shrew	*Sorex araneus*	Melanotransferrin	XP_004603294.1	75	NSSDAFSLDELR
Manatee	*Trichechus manatus latirostris*	Melanotransferrin	XP_004373762.1	75	NSSYAFTMDELR
Golden hamster	*Mesocricetus auratus*	Melanotransferrin	XP_005071744.1	75	DRSYSFTLDELR
Opossum	*Monodelphis domestica*	Melanotransferrin	XP_001381165.2	75	NSSYSFTLDELR
Horse	*Equus caballus*	Melanotransferrin	XP_005601955.1	75	NSSYAFTVDELR
Madagascar hedgehog	*Echinops telfairi*	Melanotransferrin	XP_004712393.1	75	NSSYAFTVDELR
Star-nosed mole	*Condylura cristata*	Melanotransferrin	XP_004675477.1	75	NSSYAFSLDELR
Human	*Homo Sapien*	Transferrin	AAB22049.1	33	SASD_LTWDNLK
Human	*Homo Sapien*	Lactoferrin	AAA59511.1	17	SDTSLTWNSVK

*Amino acids which differ from the human MTfp sequence are labelled in red.*

**FIGURE 3:**
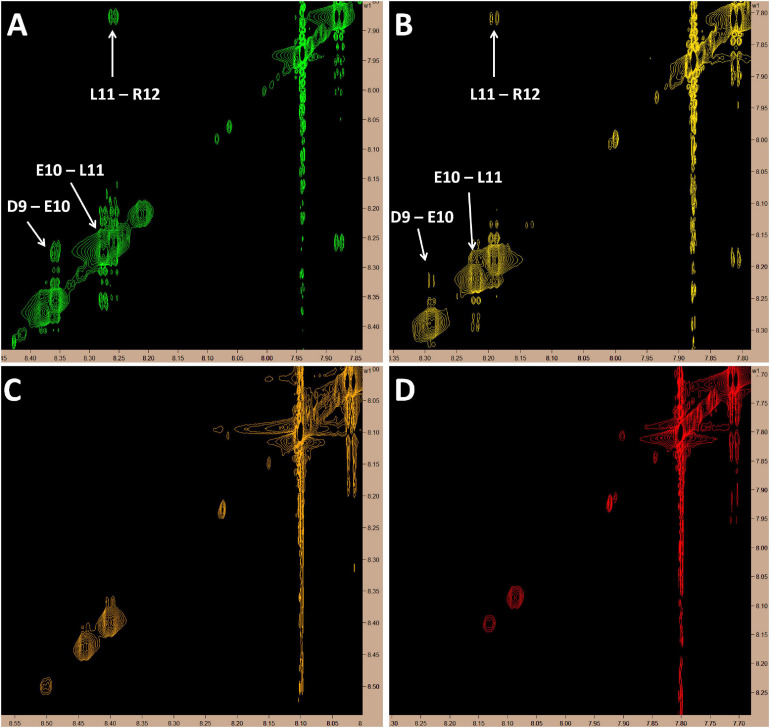
Proton NMR showing amide to amide proximity. Cross-peak signals suggestive of a helical structure in the C-terminus of MTfp can be seen at 10 and 20°C but all signals in this region fade as temperature rises; possibly due to proton exchange with solvent. All images represent 5 mM MTfp in 25 mM sodium phosphate buffer at pH 7 with 5% D_2_O. **(A)** 10°C. **(B)** 20°C. **(C)** 30°C. **(D)** 37°C.

Since this is the first known example of a peptide, derived from a larger plasma protein, being able to cross the BBB, we chose to assess the molecular dynamics of this peptide in solution over a range of temperatures (10, 20, 30, and 37°C) by proton-carbon heteronuclear multiple bond correlation spectroscopy (HMBC) and proton-proton Nuclear Overhauser Effect Spectroscopy (NOESY) nuclear magnetic resonance (NMR). As expected, based on chemical shift data from the HMBC experiments, at all temperatures, MTfp is calculated to be an unstructured coil (data not shown). However, at cooler temperatures (10 and 20°C), amide to amide NOESY signals, consistent with a helical conformation, were also observed (Figure 3).

## Discussion

Widely hailed approaches for the delivery of therapeutics to the brain often successfully demonstrate transport of the carrier or localization in an area of the brain after the BBB is disrupted by pathogenesis or by design but few, if any actually treat a CNS disease when the BBB remains intact. Different strategies have included delivery of micro-encapsulated drugs and radical methods to transiently increase the permeability of the BBB, allowing diffusion of injected drugs from the periphery into the brain (McAllister et al., 2000; Rapoport, 2000; Kinoshita et al., 2006; Pardridge, 2007a; Carpentier et al., 2016; Tsou et al., 2017). The latter approach has the additional toxicity caused by uncontrolled entry of the blood constituents into the brain and vice-versa. Traditionally, drug design has had to consider factors such as lipid solubility, charge, molecular weight and antiport action (Ohnishi et al., 1995; Patel and Patel, 2017). Drugs conjugated to small hydrophobic peptides and proteins (Pardridge, 2007a,b; Banks, 2015) or antibodies able to bind to receptors expressed on the luminal surface of the BBB (Jefferies et al., 1984; Coloma et al., 2000; Zuchero et al., 2016), have been studied. For example, MRC OX26, an antibody made against the transferrin receptor (Jefferies et al., 1984) has been used to deliver drugs to tumors in the periphery (Kratz et al., 1998) and in the brain (Lee et al., 2000; Sonoda et al., 2018). Although delivery to the brain is sometimes achievable, its use is limited due to saturation of the receptor, low dissociation rate of the antibody on the abluminal side of the BBB and consequential recycling of the receptor back to the blood (Moos and Morgan, 2001). Hyper-immunity against the carrier may also limit repeated treatments with the same drug conjugates (Tovey and Lallemand, 2011; Carrasco-Triguero et al., 2019). In addition, many of the targeted receptors are widely expressed in other tissues resulting in potential toxicity (Kratz et al., 1998). Thus, novel approaches are required to increase the survival of patients with CNS tumors and other currently untreatable brain diseases such as lysosomal storage diseases.

We have previously shown that the soluble form of mammalian iron-binding protein melanotransferrin is able to deliver small anti-cancer agents to the brain and reduce the growth of tumors therein resulting in extending the survival of the treated animals. However, there are limitations in using sMTf and other large protein carriers. These include; heterogeneity in post-translational modifications, variability in the site of cargo chemical conjugation and the adoption of different structural conformations upon conjugation that may alter the targeting moiety or the action of the drug. The other major problem is that approaches using chemical conjugates are not scalable and batch production requires standards of equivalency that are cost and time prohibitive.

In the context of the present study, the variability in conformation, glycosylation, anchoring and metal binding, presents potential complications to the use of sMTf as a robust, reproducible, clinically useful drug delivery vehicle. Therefore, to improve upon the utility of sMTf as a drug-delivery vector, we fragmented the protein in order to identify the minimally active region that retains the capability of carrying molecular cargo across the BBB. We report the identification of a 12 amino acid peptide (MTfp; DSSHAFTLDELR), derived from melanotransferrin protein, that retains the ability crosses the BBB *in vitro* and *in vivo*. Interestingly, MTfp penetrated the BBB with efficiency similar to that of a larger, known brain-targeting peptide derived from a rabies virus glycoprotein (RVGp) (Kumar et al., 2007; Alvarez-Erviti et al., 2011). Using a novel stable isotopic liquid chromatography mass spectrometry methodology to measure the transport of MTfp into the brain, we have reported elsewhere the initial rate of entry as 2.1 nM/min (Singh et al., 2020). Structural studies were then carried out to identify unique features of MTfp that enable it to retain the ability to cross the BBB and also differentiate it from its phylogenetic cousins such as Tf and Lf, which do not efficiently cross the BBB. MTfp (DSSHAFTLDELR) is well-conserved through evolution (Table 3). There is perfect sequence identity among most primates and strong conservation among many other mammals. Divergence is typically restricted to the first five residues (DSSHA). When there is a substitution, the altered amino acid is often physiochemically similar and therefore, potentially benign. The lack of variability in the C-terminal portion of MTfp suggests that this region is most important for receptor binding. This supposition is supported by the facts that human MTfp was identified by transcytosis across a model of the bovine BBB and *in vivo* transcytosis is observed in mice. Both bovine and murine MTfp homologues differ from the human version by two amino acids; both near the N terminus (Table 3). MTfp shows significantly reduced sequence identity with analogous peptides from the close MTf relatives, Tf or Lf (Table 3), this feature potentially explaining the unique ability of MTf for BBB transcytosis compared to other members of the transferrin family. The three dimensional structure of MTf, co-crystallized with the Fab portion of the anti-MTf antibody SC57.32, was only recently determined (Hayashi et al., 2021). This crystal structure confirms our analysis that the DSSHAFTLDELR peptide is not only exposed on the surface, but that it extends outward from the structure and forms part of the epitope for the antibody used in the crystallization. This implies DSSHAFTLDELR would be available as a suitable docking region for receptors.

Furthermore, we present preliminary evidence that MTfp is able to adopt a helical conformation (Figure 3). Secondary structure is unusual in such a small peptide; however, our experimental data suggests that MTfp has the propensity to adopt a transient helical character, possibly driven by repulsion between the neighboring acidic residues. Perhaps this tendency toward a helix, similar to its conformation while part of sMTf, reduces the energetic cost of peptide-receptor binding and contributes to the peptide’s unique transcytosis potential.

Designing efficient “vectors” (antibodies, protein carriers, viruses, nanoparticles) to navigate and deliver therapeutics across the BBB, in a controlled and non-invasive manner, remains one of the key goals of drug development for brain diseases. Drug design for CNS diseases is constrained by factors such as lipid solubility, charge, molecular weight, and the antiport action of specific transporters. Many methods developed to enhance the delivery of drugs to treat brain diseases have failed to provide significant improvements to long-term survival. Drugs conjugated to small hydrophobic peptides, proteins, or antibodies are able to bind to receptors expressed on the luminal surface of the BBB, however, without accumulation in the brain the resulting efficacious consequences on disease are often severely lacking. Unfortunately, these approaches are often limited due to saturation of the receptors, low dissociation rate on the abluminal side of the BBB, and recycling of the receptor back to the lumen. In addition, many targeted receptors are widely expressed in tissues other than the brain, resulting in potential toxicity and reduced transport to the brain because of competition in peripheral tissues. The data on the identification of a MTf-derived 12 amino acid peptide that retains its ability to cross the BBB and enter the intracellular compartment of resident neurons, astrocytes and microglia may begin to address many of these existing limitations.

## Data Availability Statement

The original contributions presented in the study are included in the article/Supplementary Material, further inquiries can be directed to the corresponding author/s.

## Ethics Statement

The animal study was reviewed and approved by the University of British Columbia, Animal Care Committee, under the guidelines of the Canadian Council for Animal Care.

## Author Contributions

BAE, CSBS, and WAJ wrote the manuscript. WAJ conceived the project and conducted the analysis leading to the identification of MTfp. All authors contributed to the manuscript, revised it, and approved the final version.

## Conflict of Interest

Bioasis Technologies Inc., (BTI) is a University of British Columbia start-up company. TZV, RG, and MMT were employees and equity holders in BTI at the time this work was undertaken. WAJ was the founding scientist and an equity holder in BTI at the time this work was undertaken. The funding sources had no influence in the study design, data collection, analysis or interpretation of data, in the writing of the manuscript or on the decision to submit for publication. The remaining authors declare that the research was conducted in the absence of any commercial or financial relationships that could be construed as a potential conflict of interest.

## Footnotes

1 https://www.uniprot.org/uniprot/P08582
