# Supplementary material for: Discovery of a Highly Conserved Peptide in the Iron Transporter Melanotransferrin that Traverses an Intact Blood Brain Barrier and Localizes in Neural Cells
Source: Front Neurosci. 2021 Jun 2;15:596976. doi: 10.3389/fnins.2021.596976 (PMC8212695; doi:10.3389/fnins.2021.596976)
Supplement: Supplementary file 1 [file Table_1.pdf]

Supplemental Table 1: Volume fluorescence associated with MTFp BBB transcytosis.

Animal ID	Brain Region	Field	TTV (μm^3)	BCV	BPV	TTAV	TAV_{BC}	TAV_{BP}	VTA_{BVP}
1.1 (PBS)	Cerebral Cortex	1	2.55E+05	897.57	2.54E+05	192.13	11.56	180.57	7.10E-04
	Cerebral Cortex	2	2.55E+05	514.74	2.55E+05	129.76	3.79	125.97	4.94E-04
	Cerebral Cortex	3	2.55E+05	1072.58	2.54E+05	152.17	7.46	144.71	5.69E-04
	Cerebral Cortex	4	2.55E+05	727.75	2.55E+05	123.47	5.44	118.03	4.64E-04
	Cerebral Cortex	5	2.55E+05	578.73	2.55E+05	139.00	0.44	138.56	5.44E-04
	Cerebral Cortex	6	2.55E+05	578.73	2.55E+05	139.00	0.44	138.56	5.44E-04
	Cerebral Cortex	7	4.79E+05	1649.56	4.78E+05	290.08	5.89	284.19	5.95E-04
	Cerebral Cortex	8	4.79E+05	1058.52	4.78E+05	215.69	9.73	205.96	4.31E-04
	Cerebral Cortex	9	4.79E+05	1978.99	4.77E+05	289.83	10.10	279.73	5.86E-04
	Cerebral Cortex	10	4.79E+05	1221.99	4.78E+05	268.28	10.71	257.57	5.39E-04
	Cerebral Cortex	11	4.79E+05	1333.45	4.78E+05	292.84	48.39	244.45	5.11E-04
1.2 (PBS)	Cerebral Cortex	1	2.41E+05	2376.25	2.38E+05	205.17	17.37	187.80	7.88E-04
	Cerebral Cortex	2	2.41E+05	2240.13	2.39E+05	343.29	108.66	234.63	9.83E-04
	Cerebral Cortex	3	2.41E+05	2716.72	2.38E+05	505.07	311.70	193.37	8.12E-04
	Cerebral Cortex	4	2.41E+05	686.91	2.40E+05	99.93	8.66	91.27	3.80E-04
	Cerebral Cortex	5	2.41E+05	69.71	2.41E+05	151.27	19.16	132.11	5.49E-04
	Cerebral Cortex	6	2.41E+05	325.96	2.41E+05	164.51	36.21	128.30	5.33E-04
	Cerebral Cortex	7	2.41E+05	750.74	2.40E+05	115.33	23.84	91.49	3.81E-04
	Cerebral Cortex	8	2.41E+05	323.83	2.41E+05	140.10	19.26	120.84	5.02E-04
	Cerebral Cortex	9	2.41E+05	1330.34	2.40E+05	176.80	31.37	145.43	6.07E-04
	Cerebral Cortex	10	2.41E+05	1498.67	2.39E+05	259.45	197.44	62.01	2.59E-04
1.3 (PBS)	Cerebral Cortex	1	3.49E+05	1967.35	3.47E+05	1.30	0.44	0.86	2.47E-06
	Cerebral Cortex	2	3.49E+05	1208.88	3.48E+05	312.55	45.43	267.12	7.68E-04
	Cerebral Cortex	3	3.49E+05	446.33	3.49E+05	302.79	0.00	302.79	8.68E-04
	Cerebral Cortex	4	3.49E+05	505.27	3.49E+05	351.88	0.00	351.88	1.01E-03
	Cerebral Cortex	5	3.49E+05	405.04	3.49E+05	0.68	0.54	0.13	3.84E-07
**Mean (PBS)**	5.55E-04								
**SEM (PBS)**	4.77E-05								
2.1 (RVGp-Cy5)	Cerebral Cortex	1	3.52E+05	5392.31	3.46E+05	704.40	3.28	701.12	2.02E-03
	Cerebral Cortex	2	3.52E+05	4865.26	3.47E+05	680.43	25.55	654.88	1.89E-03
	Cerebral Cortex	3	3.52E+05	4456.35	3.47E+05	519.30	34.49	484.81	1.40E-03
	Cerebral Cortex	4	3.52E+05	5210.77	3.46E+05	594.65	35.98	558.67	1.61E-03
	Cerebral Cortex	5	3.52E+05	6055.71	3.46E+05	699.63	62.48	637.15	1.84E-03
	Cerebral Cortex	6	3.52E+05	5136.29	3.47E+05	628.18	49.22	578.96	1.67E-03
2.2 (RVGp-Cy5)	Cerebral Cortex	1	4.48E+05	5125.70	4.43E+05	676.05	109.59	566.46	1.28E-03
	Cerebral Cortex	2	4.48E+05	5352.69	4.43E+05	722.45	288.55	433.90	9.80E-04
	Cerebral Cortex	3	4.48E+05	4799.19	4.43E+05	862.02	366.47	495.55	1.12E-03
	Cerebral Cortex	4	4.48E+05	5328.87	4.43E+05	831.27	286.43	544.84	1.23E-03
	Cerebral Cortex	5	4.48E+05	5324.01	4.43E+05	1014.60	46.90	967.70	2.19E-03
	Cerebral Cortex	6	4.48E+05	5793.16	4.42E+05	1097.50	528.01	569.49	1.29E-03
2.3 (RVGp-Cy5)	Cerebral Cortex	1	3.71E+05	6153.08	3.65E+05	1280.38	623.64	656.74	1.80E-03
	Cerebral Cortex	2	3.71E+05	5525.24	3.65E+05	1175.66	645.21	530.45	1.45E-03
	Cerebral Cortex	3	3.71E+05	4699.63	3.66E+05	1006.41	317.90	688.51	1.88E-03
	Cerebral Cortex	4	3.71E+05	5740.08	3.65E+05	1534.36	707.91	826.45	2.26E-03
	Cerebral Cortex	5	3.71E+05	5703.85	3.65E+05	1423.44	983.84	439.60	1.20E-03
	Cerebral Cortex	6	3.71E+05	5645.46	3.65E+05	1403.61	1057.45	346.16	9.48E-04
**Mean (RVGp-Cy5)**	1.56E-03								
**SEM (RVGp-Cy5)**	9.56E-05								
3.1 (revMTFp-Cy5)	Cerebral Cortex	1	3.35E+05	2696.06	3.32E+05	431.39	200.54	230.85	6.95E-04
	Cerebral Cortex	2	3.35E+05	2700.17	3.32E+05	453.25	305.42	147.83	4.45E-04
	Cerebral Cortex	3	3.35E+05	2105.10	3.33E+05	586.91	385.42	201.49	6.06E-04
	Cerebral Cortex	4	3.35E+05	2525.75	3.32E+05	156.36	9.23	147.13	4.43E-04
3.2 (revMTFp-Cy5)	Cerebral Cortex	1	3.56E+05	2709.04	3.54E+05	271.50	78.36	193.14	5.46E-04
	Cerebral Cortex	2	3.56E+05	2413.19	3.54E+05	362.36	175.96	186.40	5.26E-04
	Cerebral Cortex	3	3.56E+05	582.50	3.56E+05	164.83	0.20	164.63	4.63E-04
	Cerebral Cortex	4	3.56E+05	1397.83	3.55E+05	259.29	121.87	137.42	3.87E-04
	Cerebral Cortex	5	3.56E+05	2756.15	3.54E+05	290.35	109.03	181.32	5.13E-04
3.3 (revMTFp-Cy5)	Cerebral Cortex	1	2.67E+05	4195.47	2.63E+05	466.29	166.21	300.08	1.14E-03
	Cerebral Cortex	2	2.67E+05	3939.96	2.63E+05	332.55	62.79	269.76	1.02E-03
	Cerebral Cortex	3	2.67E+05	3678.81	2.64E+05	665.17	356.26	308.91	1.17E-03
	Cerebral Cortex	4	2.67E+05	2594.04	2.65E+05	52.56	6.29	46.27	1.75E-04
	Cerebral Cortex	5	2.67E+05	3855.34	2.63E+05	647.66	462.76	184.90	7.02E-04
	Cerebral Cortex	6	2.67E+05	3310.29	2.64E+05	326.27	17.75	308.52	1.17E-03
**Mean (revMTFp-Cy5)**	6.67E-04								
**SEM (revMTFp-Cy5)**	8.12E-05								

4.1 (MTFp-Cy5)	Cerebral Cortex	1	5.15E+05	5009.09	5.10E+05	1042.20	759.65	282.55	5.54E-04
	Cerebral Cortex	2	5.15E+05	5799.51	5.10E+05	440.88	106.02	334.86	6.57E-04
	Cerebral Cortex	3	5.15E+05	4303.21	5.11E+05	459.86	93.53	366.33	7.17E-04
	Cerebral Cortex	4	5.15E+05	5556.12	5.10E+05	620.31	327.82	292.49	5.74E-04
	Cerebral Cortex	5	5.15E+05	6181.36	5.09E+05	707.37	124.27	583.10	1.15E-03
	Cerebral Cortex	6	5.15E+05	6368.23	5.09E+05	856.18	470.87	385.31	7.57E-04
4.2 (MTFp-Cy5)	Cerebral Cortex	1	5.32E+05	2917.54	5.29E+05	1365.38	271.72	1093.66	2.07E-03
	Cerebral Cortex	2	5.32E+05	2381.09	5.30E+05	620.67	42.05	578.62	1.09E-03
	Cerebral Cortex	3	5.32E+05	2656.99	5.30E+05	740.36	60.17	680.19	1.28E-03
	Cerebral Cortex	4	5.32E+05	4679.75	5.28E+05	1209.97	223.99	985.98	1.87E-03
	Cerebral Cortex	5	5.32E+05	1807.86	5.30E+05	576.05	94.32	481.73	9.08E-04
	Cerebral Cortex	6	5.32E+05	2364.83	5.30E+05	906.10	303.68	602.42	1.14E-03
4.3 (MTFp-Cy5)	Cerebral Cortex	1	2.67E+05	3939.96	2.63E+05	332.55	62.79	269.76	1.02E-03
	Cerebral Cortex	2	2.67E+05	3678.81	2.64E+05	665.17	356.26	308.91	1.17E-03
	Cerebral Cortex	3	2.67E+05	3310.29	2.64E+05	326.27	17.75	308.52	1.17E-03
	Cerebral Cortex	4	2.67E+05	4195.47	2.63E+05	466.29	166.21	300.08	1.14E-03
**Mean (MTFp-Cy5)**	1.08E-03								
**SEM (MTFp-Cy5)**	1.05E-04								

TTV - Total Tissue Volume (Sum of voxels in entire reconstructed 3D field) BCV - Brain Capillary Volume (FITC voxels)

BPV - Brain Parenchyma Volume (TTV minus BCV)

TTAV - Total Test Article Volume (Total voxels of Cy5 fluorescence)

TAV_{BC} – Volume of fluorescence in Brain Capillaries (Voxels of Cy5 Co-localized with FITC voxels) TAV_{BP} - Volume of fluorescence in Parenchyma (TTAV minus TTA_{BC})

VTA_{BPV} - Volume of Parenchymal Test Article normalized to Parenchymal Volume (TAV_{BP} divided by BPV)
